# 7‐Keto Cholesterol as a Mediator in Alzheimer's Disease Pathogenesis

**DOI:** 10.1002/alz70856_105279

**Published:** 2026-01-07

**Authors:** Irundika HK Dias, Lorena Diaz‐Sanchez, Tommaso Angelini, Maura Marinozzi

**Affiliations:** ^1^ Aston University, Birmingham, West Midlands, United Kingdom; ^2^ Aston University, Birmingham, United Kingdom; ^3^ University of Perugia, Perugia, Perugia, Italy

## Abstract

**Background:**

Alzheimer's disease (AD) is a progressive neurodegenerative disorder characterized by amyloid‐beta (Aβ) plaque accumulation, tau hyperphosphorylation, and oxidative stress. Recent evidence suggests that oxysterols, particularly 7‐ketocholesterol (7‐KC) may play a pivotal role in AD pathology by exacerbating neuroinflammatory and oxidative damage. 7‐KC, a major non‐enzymatic oxidation product of cholesterol, is known to contribute to neurotoxicity through mitochondrial dysfunction, lipid peroxidation, and inflammation. Unlike other oxysterols, 7‐KC is highly reactive and has been implicated in cell death pathways relevant to neurodegeneration, including ferroptosis and autophagy dysregulation. Sulphation of 7‐KC alter its solubility, bioavailability, and interaction with cellular receptors, potentially amplifying its cytotoxic effects in neuronal and glial cells.

**Method:**

7‐Ketocholesterol‐3‐sulfate (7KCS) pyridinium salt and (25R)‐26‐hydroxycholesterol‐3‐sulfate (26HC3S) sodium salt were synthesized starting from cholesterol and diosgenin, respectively. Total sulphated oxysterols were extracted from APOE4 astrocytes and APOE4 knockout astrocytes. Briefly, cell lysates were spiked with internal deuterated standard, 7‐KCd5 and oxysterols were enriched using two‐step solid phase extraction (SPE) using a polymeric SPE column (HLB PRiME, Waters). A multiple reaction monitoring (MRM) based liquid chromatography‐mass spectrometry (LC‐MS/MS) method was developed and validated for the absolute quantification of (26HC3S) and 7KCS.

**Result:**

The LC‐MS/MS analysis successfully quantified 7KCS and 26HC3S in APOE4 astrocytes and APOE4 knockout astrocytes, revealing significantly elevated levels of sulphated oxysterols in APOE4 astrocytes compared to knockout controls.

**Conclusion:**

This data suggest a potential role in cholesterol metabolism dysregulation and neuroinflammation associated with Alzheimer's disease

